# Mare colostrum quality and relationship with foal serum immunoglobulin G concentrations and average daily weight gains

**DOI:** 10.1111/evj.14471

**Published:** 2025-01-15

**Authors:** Kirsty Gallacher, Katherine Champion, Katharine S. Denholm

**Affiliations:** ^1^ School of Biodiversity, One Health and Veterinary Medicine University of Glasgow Glasgow UK

**Keywords:** colostrum, foal, horse, mare, passive transfer, risks

## Abstract

**Background:**

Foals suffer from total failure to transfer passive immunity (TFTPI) when serum immunoglobulin (IgG) is <4 g/L, and partial failure to transfer passive immunity (PFTPI) when serum IgG is 4–8 g/L.

**Objectives:**

To explore risk factors for poor serum IgG concentration.

**Study design:**

Retrospective observational study.

**Methods:**

Data from 535 Thoroughbred foals born to 177 mares were analysed and included foal sex, birthweight, month and year of birth and colostrum Brix %. Associations between dam age and colostrum Brix (%); and between foal serum IgG and liveweight gains were also measured. Pre‐suckle colostrum samples and foal blood samples were collected (by jugular venipuncture within 12–24 h of birth). IgG was estimated in mare colostrum and foal serum using Brix refractometry and turbidimetric immunoassay, respectively. Foals were weighed within 20 h of birth; daily until 7 days of age and weekly until 130 days of age.

**Results:**

Mean foal serum IgG was 10.78 g/L (SD = 3.26) and mean mare colostrum Brix was 27.32% (SD = 5.96). A number of colostrum samples (20.9%, *n* = 112/535) measured <20% Brix and 20.4% of foals (*n* = 109/535) had serum IgG concentrations ≤8 g/L, 2.2% of foals (*n* = 12/535) had serum IgG less than or equal to 4 g/L and 18.1% (*n* = 97/535) had serum IgG between 4.1 g/L and 8 g/L. Foals had an average daily gain (ADG, kg) of 1.26 kg (SD = 0.14). Serum IgG (g/L) was associated with year of birth, birthweight (kg) and colostrum Brix (%). For every unit increase in foal birthweight (kg), there were small, significant increases in foal serum IgG (0.04 g/L, *p* = 0.04). Similarly, for every unit increased in mare colostrum Brix (%) there were small, significant increases in foal serum IgG (0.25 g/L, *p* < 0.001). Month of birth was significantly associated with colostrum Brix (%) and with average daily gains; with lower values for both outcome parameters with increasing calendar month. Increasing dam age at foaling was associated with lower colostrum Brix (%).

**Main limitations:**

Retrospective design with missing data, small convenience sample.

**Conclusions:**

Several risk factors were significantly associated with foal serum IgG and mare colostrum Brix (%) in the current work.

## 1 INTRODUCTION

Foals are said to have suffered from total failure to transfer passive immunity (TFTPI) when serum IgG concentrations are less than 4 g/L and partial failure to transfer passive immunity (PFTPI) when serum concentrations are between 4 and 8 g/L. Foals with serum IgG concentrations <4 g/L are at risk of increased morbidity^1^, ^2^, ^3^ and mortality.^4^, ^5^ In other species, FTPI has also been associated with daily liveweight gains,^6^, ^7^, ^8^ but this is not well published in horses.

IgG absorption in the foal's gut is maximal in the first 6 h of life and imperceptible by 20 h.^9^, ^10^ Insufficient quantity or quality of colostrum may also contribute to higher prevalence of FTPI in foals.^11^ Optimal IgG concentration in colostrum is around 60 g/L,^11^ although this more binary approach has more recently been replaced with something more nuanced with colostrum quality deemed: ‘very good’ if IgG concentrations exceed 80 g/L; ‘good’ if IgG concentration is between 50 and 80 g/L; ‘fair’ if IgG concentration is between 28 and 50 g/L, and ‘poor’ when IgG concentration is less than 28 g/L.^12^

Mare specific risk factors such as breed, age, prepartum nutrition and concurrent disease may affect colostrum quality.^13^, ^14^, ^15^ Foal specific risk factors may affect serum IgG concentrations such as weakness and inability to stand associated with dystocia^13^, ^16^ which may, in turn, affect quantity and timing of colostrum intake. Additionally, environmental risk factors such as excessive heat or cold stress (as shown in bovines by Olson et al.^17^) could affect colostrum composition and enterocyte IgG absorption.^18^

IgG concentrations may be measured directly in both mare colostrum and foal serum using radial immunodiffusion (RID) tests; however, this test is time consuming, expensive and technically demanding. A Brix refractometer offers a convenient, ‘stable‐side’ alternative to RID by measuring the total solids in serum and colostrum (which estimates the IgG concentration) through refraction of light (*r* = 0.71–0.84).^19^, ^20^ Optimal IgG concentration in colostrum (of 60 g/L) equates to 23%–24% on the Brix refractometer^20^ and a threshold of 7.8% is used to detect TFTPI and PFTPI.^19^ Alternatively, many equine breeding facilities prefer to employ turbidimetric immunoassay (TIA) to measure IgG concentration in neonatal foal serum as this offers a more direct measure of IgG, and has also been shown to be highly correlated with the RID reference test (*r* = 0.95)^21^ with high test sensitivity of 92% and specificity of 63%.^3^

The aims of this study were to measure associations between foal risk factors such as sex, birthweight, month and year of birth; and serum IgG concentration and to measure associations between dam age and colostrum Brix (%). Further associations between serum IgG and daily and weekly liveweight gains were measured. Finally, the relationship between mare colostrum Brix (%) and foal serum IgG concentration (measured between 12 and 24 h after birth) was explored.

## 2 MATERIALS AND METHODS

### 2.1 Study overview

In this retrospective, observational study, data were analysed from two UK stud farms between 2015 and 2022. The dataset consisted of 177 Thoroughbred mares and 535 foals (63 mares foaled at stud farm 1 and 114 mares foaled at stud farm 2). All foals were born between January and May, with the majority born in February. This was a convenience data set and a formal sample size calculation was not employed. All mares foaling on the two stud farms of interest were eligible for enrolment and no foals were excluded whether or not they were healthy and stood and suckled. The two stud farms were managed in the same way and mares could move freely between the two sites. At any given foaling event, an individual mare could foal at stud farm 1 or stud farm 2.

### 2.2 Mare management

Mares on both stud farms were vaccinated against Tetanus, Equine Influenza, Rotavirus and Equine Herpesevirus (EHV‐1/EHV‐4) during gestation. They were also treated with an ivermectin anthelmintic 1 month pre‐foaling. Mares were ‘turned out’ to grass paddocks for 8.5 h per day, in groups of 2–6 animals with fresh water provided in automated drinkers. Late gestation mares were additionally fed twice daily with 1.5–2 kg of concentrates and ad libitum hay.

### 2.3 Foal management

All foals that had serum IgG concentrations less than 8 g/L (*n* = 106) were given 1 L of plasma transfusion (ImmunoGold) and retested. Foals were given a second plasma transfusion (of a further 1 L), if their serum measurements were still less than 8 g/L after the first transfusion. Only initial (pre‐plasma transfusion) measurements were included in the dataset. Additionally, if mare colostrum for a particular foal measured less than 20% on the Brix refractometer (*n* = 44) the foal was given 200 mL of frozen stored (−20°C) mare colostrum initially, followed by a further 400 mL of frozen stored mare colostrum (high Brix colostrum >30%) from donor stud foster mares.

### 2.4 Sample collection

Pre‐suckle colostrum samples were collected from each mare, post‐foaling, by experienced stud personnel. Blood samples were collected from foals by jugular venipuncture by veterinarians within 12–24 h of birth. The concentration of IgG was estimated in mare colostrum using an optical Brix refractometer. Refrigerated serum samples were transported to an external laboratory for testing. The concentration of IgG in foal serum was measured directly by turbidimetric immunoassay (TIA, Biolis 30i Clinical Chemistry analyser).

Foals were weighed to the nearest kilogram (kg) within 20 h of birth (birthweight); every day until 7 days of age and every week thereafter until 130 days of age using an electronic weighbridge (Griffith Elder, UK).

### 2.5 Data analysis

All statistical analysis was conducted using STATA (version 17, StataCorp LLC). Histograms of each of the continuous outcome variables were plotted and visually assessed for normality. Shapiro–Wilk statistics were also calculated as a more formal test of normality. Descriptive statistics were calculated. Scatter plots and Pearson correlation coefficients were determined for serum IgG concentration (g/L) and each of dam colostrum Brix (%) and foal average daily gains (ADG, assessed between day 0 and day 130). Differences between weight measurements at each of the time frames were calculated to create boxplots. To assess the variation between Brix % for each individual mare, the range in Brix measurement for each mare was calculated. Intraclass correlation coefficients were calculated for mare and stud farm for all outcomes variables. Foals were categorised into three different weight categories: defined as 1 (<55 kg), 2 (55–60 kg), 3 (>60 kg), with roughly even numbers of foals in each category and these were compared with serum IgG concentration (<8 g/L) using chi‐squared statistics.

Univariable linear regression models were constructed with stud farm, month of birth, year of birth and foal sex as categorical predictor variables of interest for serum IgG concentration; and birthweight and colostrum Brix % as a continuous predictor variables. The predictor variables of stud farm, month of birth, year of birth, foal sex, birthweight and serum IgG were also used to construct linear regression models with ADG as the outcome of interest. Further univariable linear regression models were constructed with dam age as a continuous predictor and stud farm and month and year of birth as categorical predictor variables for colostrum Brix (%). Variables with *p* < 0.2 in univariable models were included in multivariable models, and mare was included as a random effect in the colostrum Brix (%), serum IgG and ADG models to account for repeated measures. Biologically plausible interaction terms were explored (including interactions between: foal birthweight and sex; birthweight and year and month of birth; foal sex and year and month of birth and dam age and month of birth). Confounding variables were included if model coefficients varied by >20%. Variables were excluded from the multivariable models (significance declared at *p* < 0.05) using a process of backwards stepwise elimination. Multivariable models were compared using the likelihood ratio test and Akaike Information Criteria (AIC), with a choice of final model with the lowest AIC.

## 3 RESULTS

All 535 foals were weighed within the first day of life (D0) and the mean birthweight was 57.50 kg (SD = 5.65) with a weight range of 39–74 kg. All foals were blood sampled within 24 h of life and mean serum IgG concentration was 10.78 g/L (SD = 3.26, minimum = 1.8, maximum = 22.3). Colostrum was collected from mares before foal suckling and mean colostrum Brix was 27.32% (SD = 5.96, minimum = 11, maximum = 43). There was moderate correlation between foal serum IgG concentration (as measured by TIA) and mare colostrum IgG concentration (as estimated by Brix) (*r* = 0.51) and correlation between foal serum IgG concentration and foal ADG was poor (*r* = 0.10). A number of colostrum samples (20.9%, 95% CI = 17.6–24.6%, *n* = 112/535) measured less than 20% on the Brix refractometer (equivalent to approximate IgG concentration <50 g/L). In this data set, 20.4% (95% CI = 17.0–24.0%) of foals (*n* = 109/535) had serum IgG concentrations less than or equal to 8 g/L, 2.2% of foals (95% CI = 1.2–3.9%, *n* = 12/535) had serum IgG concentrations less than or equal to 4 g/L and 18.1% (95% CI = 15.0–21.7%, *n* = 97/535) had serum IgG concentrations between 4.1 and 8 g/L.

**FIGURE 1:**
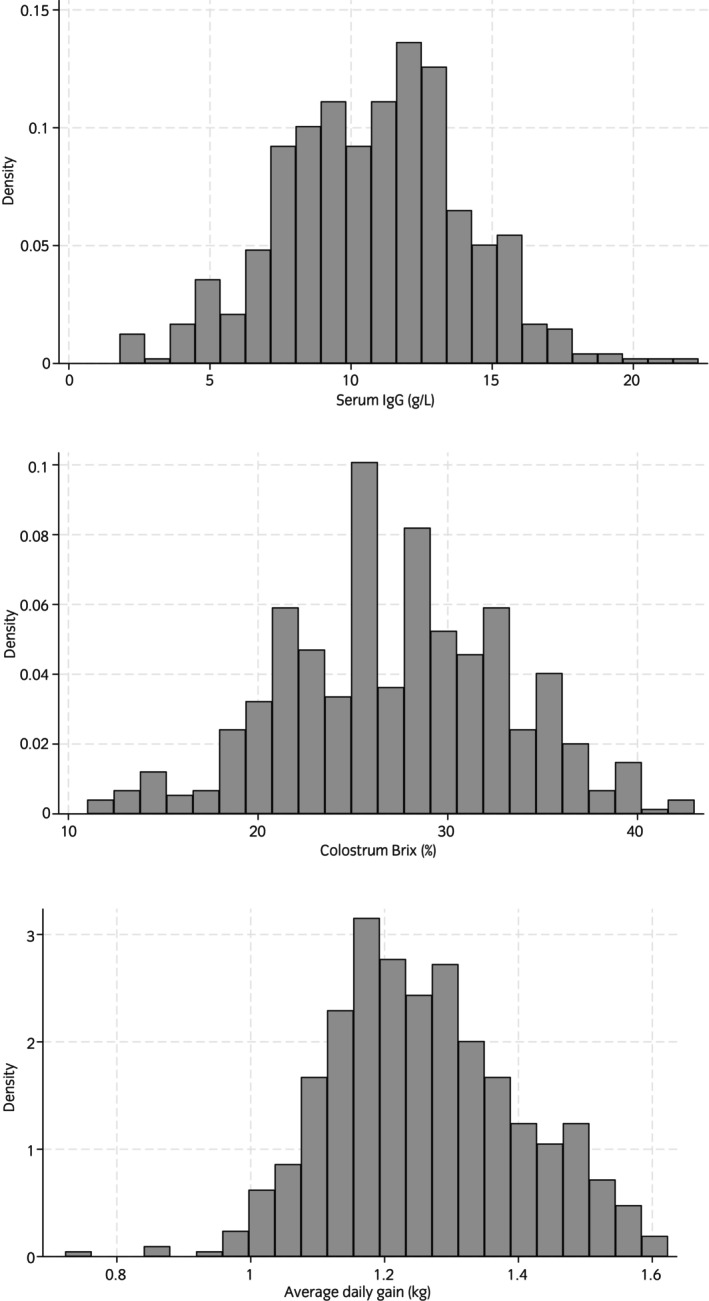
Histograms showing the distributions of the continuous outcome variable. (A) Serum IgG (g/L) measured by turbidimetric immunoassay. (B) Mare colostrum Brix (%) measured by optical refractometry. (C) Average daily gains (kg) measured from day 0 to day 130 using weigh scales for 177 mares and 535 foals between 2015 and 2022 from two UK stud farms.

Serum IgG concentration (g/L) and colostrum Brix (%) were all normally distributed on examination of histograms (Figure 1), but Shapiro–Wilk tests revealed a non‐normal distribution of ADG (kg) (*p* = 0.002); however, the decision was made to use the untransformed variable since various transformations did not improve the Shapiro–Wilk statistic.

The mean number of foals per mare (*n* = 177) was 3.03 (SD = 1.79, range 1–8 foals per mare). Dams were between 4 and 20 years of age with repeated measures for each mare in the dataset (depending on the number of foals per dam). There were 274 female foals and 261 male foals. Table 1 shows the number of foals born in each year and each month over the study period.

**TABLE 1:** Month and year of birth for 535 foals born to 177 mares from two UK stud farms collected between 2015 and 2022.

Year	2015	2016	2017	2018	2019	2020	2021	2022
Month								
January	14	10	10	7	11	6	5	2
February	16	22	17	20	17	22	18	27
March	19	23	15	24	27	21	8	20
April	12	12	15	11	14	19	17	13
May	3		4	6	3	4	5	16
Total for year	64	67	61	68	72	72	53	78

**FIGURE 2:**
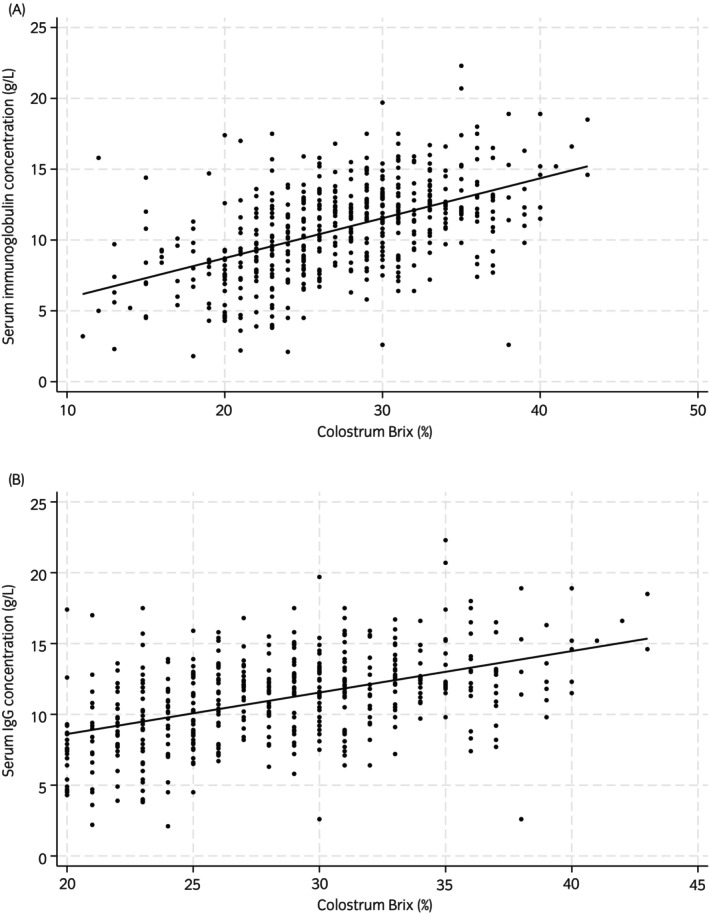
Scatter plot showing the relationship between dam colostrum Brix (%) measured by optical refractometer and foal serum IgG concentration (g/L) measured by turbidimetric immunoassay for 535 foals from 177 mares collected from 2015 and 2022 from two UK stud farms (A) All 536 mare colostrum samples (B) reduced dataset with 112 mare colostrum samples removed (since these foals would have been supplemented with extra colostrum).

**FIGURE 3:**
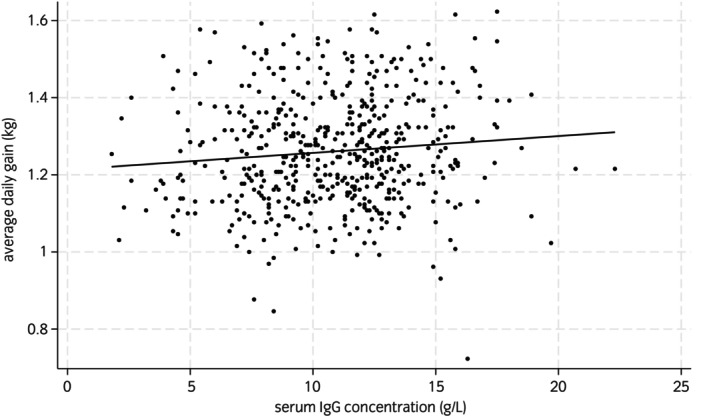
Scatter plot showing the relationship between foal serum IgG concentration (g/L) measured by turbidimetric immunoassay and average daily gains (kg) for 535 foals from 177 mares collected from 2015 and 2022 from two UK stud farms.

**FIGURE 4:**
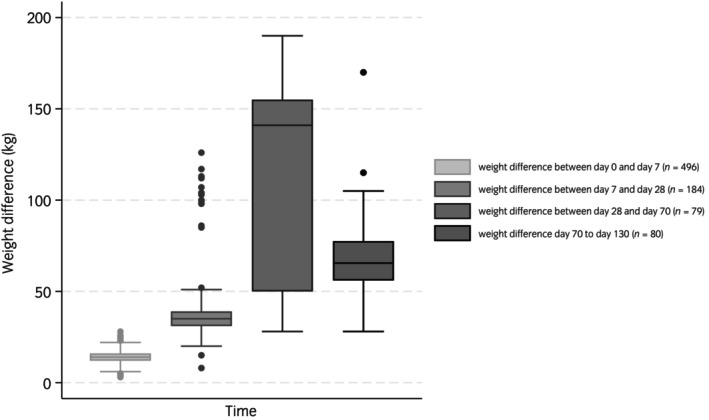
Weight differences (kg) between various time points for 535 foals from two UK stud farms measured between day 0 and day 130 of life between 2015 and 2022.

Figures 2A,B and 3 show the scatterplots between colostrum Brix and serum IgG, and serum IgG and ADG. Figure 2A presents all the data, however, in Figure 2B 112/535 (20.9%) of the mare colostrum samples were removed from the dataset to create the graph, as these colostrum samples measured <20% on the Brix refractometer, which precipitated extra colostrum supplementation to these foals (according to the stud management protocol), which would have influenced the relationship between the colostrum Brix and serum IgG measurements. In the current study 112 mare colostrum samples were low in Brix % (<20%) and of these six foals had low serum igg concentrations (≤4 g/L) with a further 50 with serum IgG concentrations 4.1–8 g/L, even with additional colostrum supplementation as part of the stud farm protocol (as described‐ Table 2). In addition, it is worth mentioning that practically equine clinicians tend to use 30% Brix to indicate ‘liquid gold’ colostrum (for storage and feeding) and that 199 samples measured ≥30% with 11 of these foals suffering from low serum IgG concentrations (≤8 g/L). Table 3 shows the weight data for 535 foals. Average daily gains were calculated by calculating the difference between day 130 and day 0 weights and dividing this difference by 130. Due to the observational nature of the study, there were many missing values for weight data (Table 1); however, all foals (*n* = 535) were weighed at day 0 and day 130 with an average daily gain (ADG, kg) of 1.26 kg (SD = 0.14, minimum = 0.72, maximum = 1.62). Mean weight gain for foals between day 0 and day 130 (*n* = 535) was 163.83 kg (SD = 18.37 kg, range 94–211 kg). Figure 4 shows the boxplot of the various weight gains throughout the rearing process with the widest variability in weight gains between day 28 and day 70. There were a number of missing data points in these observations which are indicated in Figure 4.

**TABLE 2:** Proportion of foal serum IgG samples in relation to proportion of colostrum Brix (%) samples (*n* = 535) from two UK stud farms collected between 2015 and 2022.

	Foal serum IgG category (g/L)			
Brix category	≤4	4.1–8	>8	Total
<20%	6	50	56	112
20%–23%	5	39	218	262
>23%	1	8	152	161
Total	12	97	426	

**TABLE 3:** Weight data collected from weigh scales (to the nearest kg) for 535 foals from 177 mares from two UK stud farms collected between 2015 and 2022.

Time of measurement (day)	*n*	Mean (kg)	SD (kg)	Minimum (kg)	Maximum (kg)
0	535	57.47	5.65	39.00	74.00
7	496	71.32	6.95	48.00	89.00
14	477	83.47	8.12	57.00	104.00
21	255	94.44	9.75	61.00	125.00
28	184	104.56	9.72	66.00	129.00
35	112	114.61	11.08	77.00	146.00
42	88	122.97	8.88	103.00	155.00
49	77	133.01	10.48	115.00	170.00
56	70	138.11	10.11	117.00	162.00
63	83	146.75	9.73	121.00	177.00
70	79	153.10	12.47	124.00	190.00
77	84	162.05	9.04	142.00	190.00
84	95	170.38	11.81	143.00	201.00
91	128	180.51	13.58	143.00	213.00
98	149	189.58	13.99	152.00	226.00
105	197	205.06	15.18	152.00	243.00
130	535	221.30	18.88	160.00	270.00

There was a low range in Brix measurement between individual mares of 5.0% (SD = 4.5%) and mares that produced low quality colostrum at one foaling were more likely to produce colostrum of low quality again, with much of the variability in colostrum quality attributable to mare (ICC = 0.59, SE = 0.04, 95% CI = 0.52–0.67). Additionally, much of the variability in foal ADG (ICC = 0.52, SE = 0.05, 95% CI = 0.43–0.61) and IgG concentration (ICC = 0.47, SE = 0.05, 95% CI = 0.38–0.56) was attributable to dam and (as mentioned) dam was included as a random effect term in all multivariable modelling.

Table 4 shows the comparison of the three categories of foal birthweights to a serum IgG of <8 g/L (which is the clinically relevant intervention threshold). Pearson chi‐squared test revealed that weight categories were significantly associated with serum IgG concentrations (*p* = 0.03) with mean serum IgG concentration in the <55 kg weight category of 11.17 g/L (SD = 3.19); mean serum IgG concentration in the 55–60 kg weight category of 10.75 g/L (SD = 3.21) and mean serum IgG concentration in the >60 kg weight category of 10.45 g/L (SD = 3.39). However, taking into account mare as a random effect (Table 5), there was a no statistically significant relationship (*p* = 0.07) towards foals in higher birthweight categories (of 55–60 kg) to have low serum IgG concentrations (≤8 g/L) than their lower birthweight counterparts (<55 kg), which were not different to the highest birthweight category foals (>60 kg) (*p* = 0.21). Multivariable models considered the entire dataset of 535 mares. In the multivariable models (observing foal birthweight as a continuous variable), for every unit increase in foal birthweight (kg), there were small but significant increases in foal serum IgG concentration (0.04 g/L, *p* = 0.04). Similarly, for every unit increased in mare colostrum Brix (%) there were small but significant increases in foal serum Igg concentration (0.25 g/L, *p* < 0.001). Foal sex was not significantly associated with serum IgG concentrations (see Table 6). Month of birth was significantly associated with colostrum Brix (%) and with average daily gains; with lower values for both outcome parameters with increasing calendar month. Increasing dam age at foaling was associated with lower colostrum Brix (%). Serum IgG (g/L) concentration was associated with year of birth of the foals, foal birthweight (kg) and colostrum Brix. Multivariable model residuals were found to lie within 2 standard deviations of the mean in all cases.

**TABLE 4:** Descriptive multiple comparison table to show the relationship between serum IgG concentration (<8 g/L) and foal weight categories for 535 foals born to 177 mares on two UK stud farms with Pearson chi‐squared statistic and *p* value.

	Weight category (%)						
	<55 kg		55–60 kg		>60 kg		
Serum IgG concentration (g/L)	Observed	Expected	Observed	Expected	Observed	Expected	Total
≥8 g/L	31.24 (*n* = 134/429)	28.97 (*n* = 124.3/429)	34.73 (*n* = 149/429)	34.57 (*n* = 148.3/429)	34.03 (*n* = 146/429)	36.46 (*n* = 156.4/429)	80.19 (*n* = 429/535)
<8 g/L	19.81 (*n* = 21/106)	28.96 (*n* = 30.7/106)	33.96 (*n* = 36/106)	34.62 (*n* = 36.7/106)	44.95 (*n* = 49/106)	36.42 (*n* = 38.6/106)	19.81 (*n* = 106/535)
Total	28.97 (*n* = 155/535)		34.58 (*n* = 185/535)		29.91 (*n* = 160/535)		535

*Note*: Pearson chi squared statistics = 7.31, *p* = 0.03.

**TABLE 5:** Multilevel linear regression model with serum IgG concentration as the outcome of interest and weight category as the predictor and mare a random effect term for 535 foals born from 177 mares from two UK stud farms.

Predictor	Category	Coefficient	SE	95% CI	*p* value
Intercept		0.15	0.03	0.08 to 0.21	<0.001
Weight (kg)	<55	Ref	Ref	Ref	Ref
	55–60	0.07	0.04	−0.01 to 0.15	0.07
	>60	0.06	0.04	−0.03 to 0.14	0.2

*Note*: Random effect parameters for mare: constant estimate = 0.05, SE = 0.009, 95% CI = 0.03–0.07; residual estimate = 0.11, SE = 0.008, 95% CI = 0.09–0.12.

**TABLE 6:** Final multivariable models with statistically significant risk factor variables (*p* < 0.05) for mare colostrum Brix (%) (*n* = 177) and foal serum IgG (g/L) and average daily gains (kg) (measured between day 0 and day 130) for data collected from two UK stud farms collected 2015–2022.

Outcome	Predictor	Level	Coefficient	SE	*p* value	95% CI
Colostrum Brix%^a^	Intercept		32.06	0.86	0.000	30.36–33.75
	Month of birth	January	Ref	Ref	Ref	Ref
		February	−1.95	0.63	0.002	−3.19 to −0.72
		March	−3.81	0.68	<0.001	−5.15 to −2.47
		April	−3.08	0.76	<0.001	−4.57 to −1.58
		May	−3.09	0.96	0.001	−4.97 to −1.20
	Dam age at foaling		−0.20	0.09	0.020	−0.38 to −0.03
Serum IgG (g/L)^b^	Intercept		0.04	1.40	>0.9	0.001–0.08
	Year of birth	2015	Ref	Ref	Ref	Ref
		2016	1.08	0.37	0.004	0.35–1.81
		2017	1.27	0.38	0.001	0.53–2.02
		2018	1.38	0.38	<0.001	0.64–2.12
		2019	1.20	0.38	0.002	0.45–1.95
		2020	2.01	0.38	<0.001	1.26–2.76
		2021	2.51	0.42	<0.001	1.68–3.33
		2022	3.88	0.39	<0.001	3.13–4.64
	Colostrum Brix (%)		0.25	0.02	<0.001	0.20–0.29
	Foal birthweight (kg)		0.04	0.02	0.04	0.001–0.08
Average daily gain (kg)^c^	Intercept		1.30	0.02	0.000	1.26–1.33
	Month of birth	January	Ref	Ref	Ref	Ref
		February	‐0.03	0.02	0.1	−0.058 to 0.008
		March	−0.034	0.018	0.06	−0.068 to 0.001
		April	−0.056	0.019	0.004	−0.093 to −0.018
		May	−0.047	0.024	0.05	−0.095 to −0.001

^a^ Colostrum Brix (%) multivariable model random effect parameters: estimate = 17.90, SE = 2.47, 95% CI = 13.67–23.45.

^b^ Serum IgG multivariable model random effect parameters: estimate = 2.61, SE = 0.45, 95% CI = 1.86–3.66.

^c^ Average daily gain multivariable model random effect parameters: estimate = 0.01, SE = 0.002, 95% CI = 0.007–0.014.

## 4 DISCUSSION

The objective of this study was to measure associations between foal risk factors and serum IgG concentration; as well as between dam risk factors and colostrum Brix (%). Further associations between serum IgG and daily and weekly liveweight gains were measured. Finally, the relationship between mare colostrum Brix (%) and foal serum IgG concentration (measured between 12 and 24 h after birth) was explored.

The concentration of serum IgG in foals is highly variable.^22^ Reported FTPI prevalence ranges from 2.9% and 25%.^13^, ^23^, ^24^ In a recent Italian study of 81 mares, 17.28% of foals had FTPI or PFTPI,^20^ similar to the current study where 20.40% (*n* = 109/535) of foals had serum IgG concentration ≤8 g/L. Colostrum IgG concentration also varies widely between individual mares,^22^, ^25^, ^26^ but there is a dearth of large datasets in equine published literature to fully understand individual mare effects on colostrum IgG concentration and volume. In a recent study of 80 samples, 18.52% of colostrum samples had inadequate concentrations of IgG (<60 g/L),^20^ similar to the current work where 20.93% of colostrum samples were <23%. A review article by Chapman^15^ listed many risk factors associated with mare colostrum IgG concentration including: poor maternal immune or nutritional status; premature lactation, placentitis or placental separation; advanced maternal age; maiden pregnancies; breed; and endophyte toxicity from contaminated grass or hay.^13^, ^14^ There is a dearth of published literature on risk factors for poor colostrum volume production in the mare, although stress; mastitis and udder structural damage have been suggested.^13^ Pre‐partum nutrition and seasonal effects are established risk factors for poor colostrum quality and volume in other species.^27^, ^28^ Later month foaled was associated with lower colostrum Brix % in the current work. Seasonal effects on colostrum quality and volume of first milking colostrum production have been demonstrated in cattle^27^, ^28^, ^29^ and heat stress has been suggested to affect colostrum quality and quantity in other species.^18^ A non‐peer reviewed thesis showed a positive correlation between month foaled and foal serum IgG concentration.^30^

In the current study, there was an association between month born and foal average daily gains with foals born later in the season having lower average daily gains. This is opposite to what might be expected with January seasonal temperatures lower than May temperatures, since in colder temperatures animals will require more energy to thermoregulate (demonstrated in calves by Bell et al.^31^). There was no statistically significant relationship between serum IgG concentration and foal ADG in the current work and weak correlation between these variables (*r* = 0.10).

Rampacci et al.^20^ reported that 64.3% of the foals with FTPI/PFTPI suckled colostrum of inadequate quality. It has also been reported in the literature that 67% of foals receiving low quality, and 45% of foals receiving mediocre quality colostrum have failure of passive transfer of immunity.^32^ In the current study, of the 112 poor quality colostrum samples 56 foals had FTPI (50.0%). Additionally, in the current work, mare colostrum IgG concentration was significantly associated (*p* < 0.001) with foal serum IgG concentration and correlation was moderate (*r* = 0.51). A moderate correlation (*r* = 0.54) was also observed between IgG concentrations measured by RID in foal serum and mare colostrum in other work,^20^ with poor to moderate correlation reported by various other authors.^23^, ^30^, ^33^ The highest correlation statistic between serum IgG and colostrum IgG (measured by RID) in horses was reported by Sobral et al.^12^ (*r* = 0.66).

Some work has elucidated that increasing age of the mare (>15 years) is negatively associated with colostrum IgG concentration.^34^ Increasing dam age was also negatively associated with colostrum Brix (%) in the current study; however, other literature has shown no effect.^23^ It is worth noting that in the current work only 2.24% (*n* = 12/535) were more than 15 years old at the time of foaling. Nevertheless, increasing dam age resulted in lower colostrum quality and this may have been due to comorbidities in older mare (although this was not measured). A 2020 study by Barreto et al.^35^ reported that breed influences the percentage of protein found in colostrum, suggesting that the colostrum of Quarter Horse mares may contain more protein and be less dense in energy when compared with other breeds. Another study showed that draught mares produce better quality colostrum than saddle mares.^34^, ^36^ Mare breed was not included in the current risk factor cohort since the current study included a homogenesis cohort of Thoroughbred mares. In more recent studies, no maternal variable, including breed influenced colostrum IgG concentrations.^20^, ^23^

Serum IgG concentration can be measured in foals as early as 8–12 h after birth since IgG peaks in serum at around 12 h.^37^ All foals in the current study were sampled within 24 h of birth; however, exact timing between sucking and sampling for individual foals was beyond the scope of the current work. By measuring serum concentrations in foals at 8–12 h, foals with low serum IgG concentration can be supplemented orally with colostrum prior to gut closure, preferably using stored mare colostrum, although colostrum from other species may be used with caution.^11^ Immune deficient foals may also be supplemented with fresh‐frozen hyperimmune plasma, which has been shown to be safe and effective.^38^ It has been suggested that supplementation with 1 L of plasma may increase serum IgG concentrations by 200 mg although this varies depending on clinical signs of dehydration and/or sepsis.^11^, ^39^ Serum IgG may also undergo rapid catabolism in critically ill foals^11^, ^15^ and repeated measurement of serum IgG concentration is recommended 24 h after plasma administration.

In the current study, of the 112 colostrum samples with low Brix (<20%), 56 of these resulted in foals with serum IgG concentrations ≤8 g/L (indicating partial or complete FTPI), which suggests that further high quality colostrum supplementation for these foals (as per stud farm protocol) may not be happening promptly enough to allow for absorption through the foals' neonatal enterocytes before they become impermeable to large IgG molecules. Alternatively supplemented serum IgG may be undergoing catabolism in these foals, although morbidity and mortality estimations were beyond the scope of the current data.

Although in the multivariable modelling, increasing foal birthweight was associated with overall increasing serum IgG concentration, when weight categories were investigated, foals in the higher birthweight categories tended to have numerically lower serum IgG concentrations (see Table 4 where 44.95% of foals in the over 60 kg category had serum IgG concentrations less than 8 g/L vs. 19.81% of foals in the under 55 kg category). Clinicians tend to be concerned for the overall health status of lower birthweight foals, but these findings may raise increased concern for large birthweight foals and for due diligence in testing serum IgG levels in all foals.

The mean birthweight of foals in this study was 57.5 kg (SD 5.65), with a range of 39–74 kg. This information is of interest as it demonstrates a higher mean birthweight for Thoroughbred foals than previously recorded in the literature, with mean foal weight of 49.6 kg in the 1970's,^40^ following an upward trajectory through subsequent decades (Table S1). Noting this increase in mean birthweight is relevant for colostrum management since larger foals will require a higher volume of quality colostrum in order to confer passive immunity and clinical decision making regarding therapeutic dosage.

### 4.1 Study limitations

These data were observational using information from two commercial UK stud farms and analysed retrospectively. As such researchers had little control over the measured outcomes or other risk factors for poor serum or colostrum IgG concentration including injury, nutrition and disease processes and there was no prior sample size calculation. The convenience nature of the sample from only two related stud farms may also limit the external validity of the results. The IgG concentration in mare colostrum was indirectly estimated using Brix refractometry and would have been more accurately compared with serum IgG concentration using a direct measure of IgG concentration (using RID testing). Growth rates for each time frame were estimated but there were many missing values in this observational dataset which may have influenced results. Dam age was available for analysis, but no information on whether the mares were primiparous or multiparous was available and parity may have been associated with mare colostrum quality and foal serum IgG concentration. Although the initial serum IgG is recorded in the dataset, a number of foals (*n* = 106) were supplemented with additional IgG through plasma transfusion (if their serum IgG was less than 8 g/L) and this may have impacted average daily gains (although serum IgG was only significantly associated with ADG at the univariable level *p* = 0.02 but not in the final models). ADG was calculated by dividing the ADG difference between day 0 and day 130 but some foals may not have been weighted at precisely day 130 and this may have influenced results. A minority of foals were also supplemented with extra colostrum if their own mare's colostrum was lower than 20% and this could have influenced the measured relationships between serum IgG and colostrum Brix although when the reduced dataset (removing the foals where their dam's colostrum was less than 20%; approximately 20% of all mare colostrum) did not change the significance of the relationship between the two variables and reduced the Pearson correlation coefficient from 0.51 to 0.47 (Figure 2).

## 5 CONCLUSIONS

Several risk factors were significantly associated with foal serum IgG and mare colostrum Brix (%) in the current work. Foal serum IgG concentration was associated with colostrum Brix %, year of birth and foal birthweight. Later month foaled and older dams were associated with lower colostrum Brix % in the current work, and mares that produced low quality colostrum were more likely to do so in future (cv = 0.13). Mare colostrum IgG concentration was significantly associated with foal serum IgG concentration. Foals born later in the season had lower average daily gains; however, there no significant association between foal serum IgG and ADG. In the current study, of the 112 colostrum samples with low Brix (<20%), 56 of these resulted in foals with serum IgG concentrations ≤8 g/L (indicating partial or complete FTPI), which suggests that further high‐quality colostrum supplementation for these foals needs to happen more promptly in practice. Clinicians should be diligent in testing serum IgG levels in all foals and ensure that foals with higher birthweights receive adequate colostrum since there has been an upward trend in Thoroughbred foal birthweights.

## FUNDING INFORMATION

No specific funding was received for this work.

## CONFLICT OF INTEREST STATEMENT

The authors declare no conflicts of interest.

## AUTHOR CONTRIBUTIONS

**Kirsty Gallacher:** Conceptualization; project administration; investigation; writing – review and editing. **Katherine Champion:** Formal analysis; data curation; validation; visualization; writing – review and editing. **Katharine S. Denholm:** Methodology; investigation; formal analysis; supervision; writing – original draft; visualization; conceptualization.

## DATA INTEGRITY STATEMENT

K. Denholm had full access to all the data in the study and takes responsibility for the integrity of the data and the accuracy of the data analysis. K. Denholm is responsible for data integrity.

## ETHICAL ANIMAL RESEARCH

Data were analysed under University of Glasgow ethics licence number EA56/23.

## INFORMED CONSENT

Stud owners gave general consent for use of medical records for research.

## Supporting information


**Table S1.** Foal birthweight data from published literature from 1966 to present, indicating an upward trajectory in Thoroughbred foal birthweights.

## Data Availability

The data that support the findings of this study are openly available from University of Glasgow Enlighten https://researchdata.gla.ac.uk/cgi/search/advanced under reference number 1663.
