# Supplementary material for: Mare colostrum quality and relationship with foal serum immunoglobulin G concentrations and average daily weight gains
Source: Equine Vet J. 2025 Jan 15;57(4):904–14. doi: 10.1111/evj.14471 (PMC12135750; doi:10.1111/evj.14471)
Supplement: Supplementary file 1 — Table S1. Foal birthweight data from published literature from 1966 to present, indicating an upward trajectory in Thoroughbred foal birthweights. [file EVJ-57-904-s001.pdf]

Equine Veterinary Journal BEVA

Table S1: Foal birthweight data from published literature from 1966 to present, indicating an upward trajectory in Thoroughbred foal birthweights.

Year	Mean weight (kg)	Weight SD (kg)	Weight range (kg	Foals (n)	Country	Farms (n)	Author
**1966**	50				UK		Rossdale, 1966
**1968-1973**	51.2	6.2	13.6-70.8	796	UK		Platt, 1978
**1976**	49.6	0.45		144	UK		Rossdale, 1976
**20 years**	53.6	5.2	39.0-67.2	128	USA (Florida)		Kavazis and Ott, 2003
**1977-2007**	55.9	6.1		1374	USA, UK, IRE	34	Kocher and Burnton Staniar, 2013
**2006**	55.2	7.1	29-75	348	AUS (NSW)	1	Elliot et al., 2009
**2007-2019**	55.3	6.5		934	UK	6	Pagan et al., 2023
**2009-2019**	54	0.3	37-72	495	ARG	7	Pellegrini et al., 2024
**2013-2018**	56.5	6.6		801	USA (KY)	12	Pagan et al., 2023
**2013-2019**	53.9	6.6		1327	AUS	1	Pagan et al., 2023
**2015-2022**	57.5	5.7	39-74	535	UK	2	Current study

References:

Elliott C, Morton J, Chopin J. Factors affecting foal birth weight in Thoroughbred horses. Theriogenology. 2009;71:683-689. doi: 10.1016/j.theriogenology.2008.09.041. Epub 2008 Nov 5. PMID: 18980778.

Kavazis AN, Ott EA. Growth Rates in Thoroughbred Horses Raised in Florida. J Equine Vet Sci. 2003;23:353-357. https://doi.org/10.1016/S0737-0806(03)01024-4.

Kocher A, Burton Staniar W The pattern of thoroughbred growth is affected by a foal's birthdate. Livestock Science. 2013;154:204-214. https://doi.org/10.1016/j.livsci.2013.03.008.

Pagan JD, Phethean E, Caddel S, McFaull M, Bonner P, Nowara D, Perriam W, Huntington PJ. Factors affecting birth weights in Thoroughbred foals born in Kentucky, United Kingdom, and Australia. J Equine Vet Sci. 2023;124. https://doi.org/10.1016/j.jevs.2023.104419.

Pellegrini AG, Paz S, Trigo P, Losinno L, Piccardi MB. Modeling growth curves in Thoroughbred foals raised on pasture in Argentina. Livestock Sci. 2024;285:105501.http://dx.doi.org/10.1016/j.livsci.2024.105501.

Platt H. Growth and Maturity in the Equine Fetus. J Royal Soc Med 1978;71:658-661.

Rossdale PD. Fellowship thesis Royal College of Veterinary Surgeons. 1966

Rossdale PD. Perinatal development: A Clinicians View of Prematurity and Dysmaturity in Thoroughbred Foals. Proc Royal Soc Med. 1976;69:631-632.
